# Entropy, Carnot Cycle and Information Theory, Part 2

**DOI:** 10.3390/e27050493

**Published:** 2025-05-02

**Authors:** Mario Martinelli

**Affiliations:** Dipartimento di Elettronica Informazione e Bioingegneria, Politecnico di Milano, 20133 Milano, Italy; mario.martinelli@polimi.it

**Keywords:** Carnot cycle, entropy, Lagrange parameter, Schrodinger equations, information theory

## Abstract

This second part of this companion paper the Carnot cycle is analyzed trying to investigate the similarities and differences between a framework related to thermodynamics and one related to information theory. The parametric Schrodinger equations are the starting point for the framing. In the thermodynamics frame, a new interpretation of the free energy in the isothermal expansion and a new interpretation of the entropy in the adiabatic phase are highlighted. The same Schrodinger equations are then applied in an information theory framework. Again, it is shown that a cycle can be constructed with a diagram that presents the Lagrange parameter and the average codeword length as coordinates. In this case the adiabatic phase consists of a transcoding operation and the cycle as a whole shows a positive or negative balance of information. In conclusion, the Carnot cycle continues to be a source of knowledge of complex systems in which entropy plays a central role.

## 1. Forward

In the first paper [1] with the same title the Carnot cycle was analyzed starting from the partition function Z, a function that is shared by both thermodynamics (as underlined by Reiss [2], in thermodynamics it is at the base of the “transformation theory”) and information theory (Jaynes [3] pointed out the link between the partition function and the capacity of an communication system). More precisely, in [1] the isothermal expansion of the Carnot cycle was analyzed in the light of the Kullback–Leibler distance even in the presence of a perturbation introduced in the isothermal phase by an information input.

Everything arises from the observation that the two *founding equations* of thermodynamics (as reported by Schrodinger in [4] (reprint of the first 1946 publication)) and so called in the following *parametric Schrodinger equations,* or *PSEs*,

(1) $$\langle f\rangle =-\frac{d}{d\lambda }lnZ\left(\lambda \right)\;$$

(2) $$S=klnZ\left(\lambda \right)+k\lambda \langle f\rangle$$

can also be interpreted with the formalism of information theory. In (1) and (2) *S* is the entropy, *k* is the Boltzmann constant, λ the Lagrange multiplier, *Z* is the partition function of the process under examination defined as

(3) $$Z=\sum_{i=1}^{A}e^{-\lambda x_{i}}$$

where *A* represents the *“dimension of the considered ensemble of distinguishable groups labelling a random variable”* [5]: in quantum mechanics or statistical mechanics (and hence in thermodynamics), this ensemble is the set of all possible energy eigenvalues (i.e., either of the harmonic oscillator or of the molecules of the gas considered); in information theory, the set (obviously much smaller) represents either the number of characters of the considered alphabet or the number of words of the considered vocabulary. In this context $x_{i}$ is a generic “quality” of the “distinguishable group” such as the *energy value* in thermodynamics or the *character duration* or the *length of a codeword* in information theory and the index “*i*” is the numeration of the distinguishable groups. Finally, $\langle f\rangle$ is the expectation value of $x_{i}$.

Since its origin, information theory has had a relationship with thermodynamics [5]. Shannon, who, in his fundamental work [6] on formulating the 2nd theorem, admits that: *“The form H will be recognized as that of entropy as defined in certain formulation of statistical mechanics”*. It is Jaynes, however, in a couple of papers published in 1957 [7] and 1959 [3], who pointed out the existence of a deep relationship between thermodynamics and information theory, which share the concept of “partition function”: “*…the basic mathematical identity of the two fields* [thermodynamics and information theory] *has had, thus far, very little influence on the development of either. There is an inevitable difference in detail, because the applications are so different; but we should at least develop a certain area of common language…we suggest that one way of doing this is to recognize that the partition function, for many decades the standard avenue through which calculations in statistical mechanics are “channelled” is equally fundamental to communication theory*”.

However, in the paper of 1959, Jaynes pointed out some “singularities” of information theory that are not found in thermodynamics and therefore prevent the development of simple parallelism. In particular, Jaynes notes that Shannon’s theorem 1, the theorem which defines the channel capacity, does not present an equivalent in thermodynamics. He says “*It is interesting that such a fundamental notion as channel capacity has not a thermodynamic analogy*”. And he continued by underlining that: “*the absolute value of entropy has no meaning, only entropy differences can be measured in experiments. Consequently the condition that H is maximized, equivalent to the statements that the Hemholtz free energy function vanishes, corresponds to no condition which could be detected experimentally*”.

The above singularities inhibit extension to information theory of all the fundamental axioms of thermodynamics, including the Carnot theorem that, as a consequence, does not present, in our opinion, an exact parallel in information theory. Essentially, the Carnot theorem is a *conservation theorem* because it says that, during a reversible thermodynamics cycle finalized to extract work by exchange of heat from two reservoirs at different temperatures, the entropy of the cycle is conserved. This was exactly noted by Carnot who, in his monograph (as translated and edited by E. Mendosa [8]) of 1824, says: *“The production of motion in steam-engine is always accompanied by a circumstance on which we should fix our attention. This circumstance is the re-establishing of equilibrium in the caloric; that is, its passage from a body in which the temperature is more o less elevated, to an other in which it is lower”*. If the term “caloric” could be interpreted as “entropy” (Carnot used two terms for heat: “chaleur” and “calorique” and from the context we can deduce that with the second term he indicated a concept close to the current “entropy” [8]), we have here the first claim of the “entropy conservation” principle in a reversible Carnot cycle. He also observed that: “*The production of motive power is then due in steam-engines not to an actual consumption of caloric, but to its transportation from a warm body to a cold body, that is, to its re-establishment of equilibrium*”. It is worth noting that, in the same sentence, Carnot notes that the cycle is finalized to the production of motion. The absence of such production posed some problems, as pointed out by William Thomson (one of the first commenters of Carnot’s book) in 1848 [9]: “*When “thermal agency” is thus spent in conducting heat through a solid, what becomes of the mechanical effect which it might produce? Nothing can be lost in the operations of nature—no energy can be destroyed. What effect then is produced in place of the mechanical effect which is lost? A perfect theory of heat imperatively demands an answer to this question: yet no answer can be given in the present state of science…*”. The answer to Thomson’s observation came from the German scientist Rudolf Clausius (the scientist that introduced the term “entropy” in 1865 [10]) who in 1850 was already aware that heat is not only inter-convertible with mechanical work but in fact actually may *“consist in a motion of the least parts of the bodies”* and with this latter conclusion he formulated the equivalence of heat and work.

The question posed in 1848 by William Thomson has similarities to the one that comes naturally when we use PSEs to “export” a Carnot cycle in information theory. In fact, by using PSEs it is possible to reproduce a Carnot cycle where entropy/information is conserved but the question that also rises spontaneously in this case is: *“What is produced in this cycle since no mechanical work is produced?”*.

In the following we will try to give an answer to this question. We will first propose a synthesis of the Carnot thermodynamic cycle, already developed in part I, and then we will try to develop above all the vision from the point of view of the theory of information.

## 2. The Thermodynamics Carnot Cycle in the Light of the “Parametric Schrodinger Equations”

As already mentioned in the Introduction, in thermodynamics the variable $x_{i}$ is the energy of the molecules of the gas,$\;\epsilon_{i}$, and the function $\langle f\rangle$ becomes the internal energy $\langle U\rangle$. Hence, from (1) we have

(4) $$\langle U\rangle =\sum_{i}p_{i}\epsilon_{i}=\frac{1}{Z}\sum_{i}e^{-i\frac{\epsilon_{i}}{kT}}\epsilon_{i}$$

where we have explicated the Lagrange multiplier by 1/kT, the inverse of the absolute temperature times the Boltzmann constant, and the probability $p_{i}$ of finding the system in the distinguishable group of index “i”.

M. Tribus [11] provides an important interpretation of the variation of internal energy (4) that allows us to better understand the meaning of expressions (1) and (2). Starting from relation (4) Tribus derives that

(5) $$d\langle U\rangle =\sum_{i}\epsilon_{i}{dp}_{i}+\sum_{i}p_{i}{d\epsilon }_{i}$$

and he assigns to the first sum after the equals sign the meaning of “*change of heat, dQ*” and to the second term the meaning of “*negative change of work, −dW*” in order to obtain the classical relationship

(6) $$d\langle U\rangle =dQ-dW\;$$

and comments: “A change of the type dW, causes no change uncertainty *S*, since $p_{i}$ are unchanged. A change of the type dQ, is associated with a change in *S*”. Tribus accompanies these considerations with three illustrations [11] of the distributions of $\epsilon_{i}$ and $p_{i}$ and the product of $p_{i}\epsilon_{i}$.

It is interesting to note that in the case where the thermodynamic system considered is a “black body”, the distribution of $p_{i}\epsilon_{i}$ is nothing other than the *single-mode Planck’s law*.

Thermodynamic potentials can also be derived from the PSE. The most direct of these is the Helmholtz potential or “free energy” which is connected to the partition function (see [1] and the references contained therein) by the relation

(7) $$F=-{kT}_{2}lnZ$$

or, by introducing the partition function expression (3),

(8) $$F=-{kT}_{2}ln\left[\sum_{i=1}^{A}e^{-\frac{1}{kT_{2}}\epsilon_{i}}\right]=\sum_{i=1}^{A}\epsilon_{i}$$

We thus obtain a new interpretation of free energy *F*, a relevant thermodynamics potential in all thermodynamic cycles in which work is produced: *it is simply the sum of all possible energy levels of the thermodynamic system under consideration*.

By using (7), (4) becomes

(9) $$S=klnZ\left(\lambda \right)+k\lambda \langle f\rangle =-\frac{F}{T_{2}}+\frac{\langle U\rangle }{T_{2}}$$

**Figure 1:**
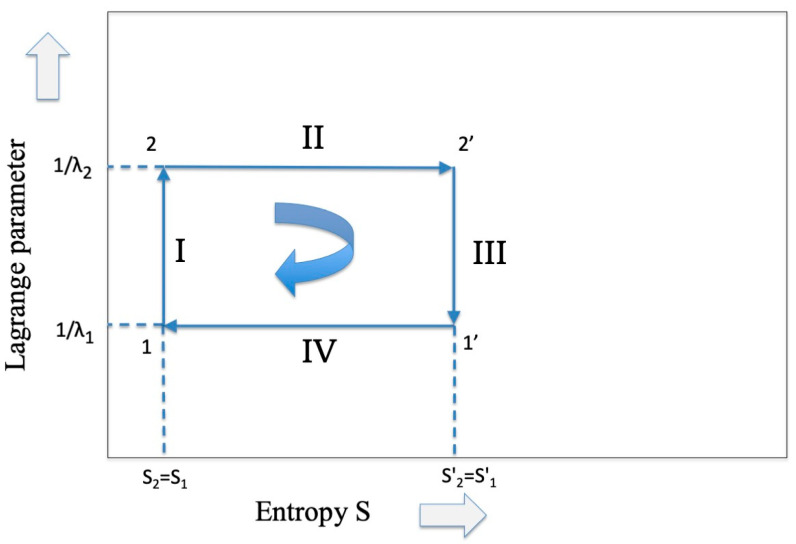
The representation of the Carnot cycle which is common to both thermodynamics and information theory.

Starting from this relation, let us study the Carnot cycle in the ST representation (see reference [1], reported again for convenience as Figure 1, where the inverse of the Lagrange parameter has been adopted) for the isothermal (II and IV) and the adiabatic branches (III and I).

As far as the *isothermal expansion* (from point 2 to 2′ in Figure 1) is concerned, we know [1] that the flux of energy coming from the high-temperature reservoir is converted only into free energy (work) while the internal energy remains constant. Hence, we have that

(10) $$\Delta S_{2}=S_{2}^{\prime }-S_{2}=-\frac{F_{2}^{\prime }}{T_{2}}+\frac{F_{2}}{T_{2}}=\frac{∆F_{2}}{T_{2}}$$

or, from (8),

(11) $$∆S_{2}=kT_{2}\left(ln\sum_{i=1}^{A}e^{-\frac{\epsilon_{2^{\prime }}}{kT_{2}}}-ln\sum_{i=1}^{A}e^{-\frac{\epsilon_{2}}{{kT}_{2}}}\right)=\sum_{i=1}^{A}\left(\epsilon_{2^{\prime }}-\epsilon_{2}\right)$$

an expression which evidences that the increase in entropy is a consequence of the sum (over the whole ensemble) of the *energy differences* between the end and the beginning of the expansion. Note that the ensemble dimension *A* remains constant.

**Figure 2:**
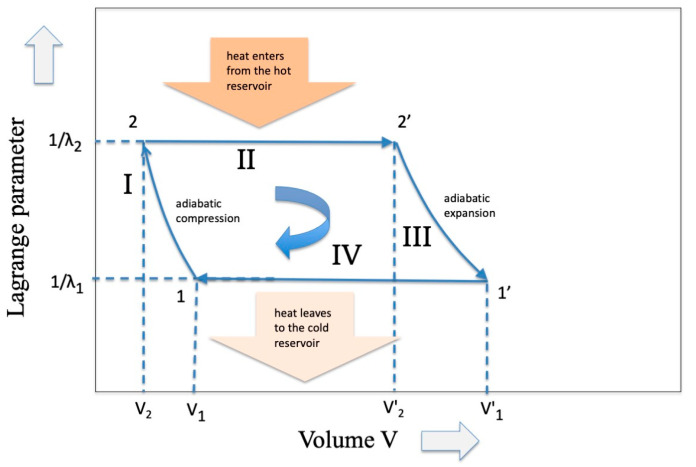
The classic representation of the Carnot Cycle in the variables Temperature (inverse of the Lagrange parameter) and Volume of an ideal gas.

But *why are these energy eigenvalues different*? To find the answer we must refer to a “hidden” variable that does not appear in Equations (1) and (2), that is, the “volume V”, a variable that enters into the Carnot cycle when a gas is involved (see Figure 2). In the expansion phase a work W is delivered through the relation

(12) $$W_{2}=-\int_{V_{2}T_{2}}^{V_{2}^{\prime }T_{2}}PdV=-RTln\frac{V_{2}^{\prime }}{V_{2}}$$

where *P* is the pressure and *R* the gas constant. Hence, since

(13) $$W_{2}=∆F_{2}=-{kT}_{2}lnZ_{2}^{\prime }+{kT}_{2}lnZ_{2}=kT_{2}ln\frac{Z_{2}}{Z_{2}^{\prime }}\;$$

it is evident that the different energy eigenvalues of (11) are the direct consequence of the differences in the partition functions, whose ensemble changed because the volumes changed (and not the temperature which remains stable at $T_{2}$).

The isothermal phase II is inversely replicated in the compression phase IV (from points 1′ to 1 in Figure 1) at the lower temperature $T_{1}$, and the sum of the two entropy changes, giving zero. Hence,

(14) $${∆S}_{2}-∆S_{1}=\frac{∆F_{2}}{T_{2}}-\frac{∆F_{1}}{T_{1}}=0\;⟶∆F_{2}T_{1}=∆F_{1}T_{2}$$

or, from (13),

(15) $$W_{2}=\frac{T_{2}}{T_{1}}W_{1}$$

a result that allows obtaining the *efficiency of the Carnot cycle*

(16) $$\eta =\frac{W_{2}-W_{1}}{W_{2}}=1-\frac{W_{1}}{W_{2}}=1-\frac{T_{1}}{T_{2}}$$

As far as *the adiabatic phase III* (from points 2′ to 1′ in Figure 1) is concerned, we have that the entropy is conserved, thus

(17) $$∆S\prime =S_{1}^{\prime }-S_{2}^{\prime }=-\sum_{i=1}^{A\prime }p_{1i}lnp_{1i}+\sum_{i=1}^{A}p_{2i}lnp_{2i}=0\;$$

where we introduce the new ensemble dimension *A′* because we are now at temperature *T_1_*.

That is, in the adiabatic phase we observe a complete change of the ensemble considered because both the temperature and the volume vary and therefore the size of the ensemble, the partition function, the set of energy eigenvalues, and the probability distribution change. In entropic terms, even if it is true that the final entropy remains equal in value to the initial entropy, the two values are obtained *as a result of a different ensemble average*.

In phase III an amount of work is delivered whose expression is

(18) $$∆W=-\int_{v_{2}^{\prime },T_{2}}^{v_{1}^{\prime },T_{1}}PdV$$

and by introducing the relations

(19) $$c_{v}={\left(\frac{dU}{dT}\right)}_{v}$$

and

(20) $$dQ=dU+PdV\;\Rightarrow PdV=dQ-c_{v}dT\;\Rightarrow for\;the\;adiabatic\;process=-c_{v}dT.$$

we obtain

(21) $$∆W={-c}_{v}\left(T_{1}-T_{2}\right)$$

These terms represent the work given by the external work reservoir in the system (by the system on the external work reservoir in symmetric phase I, from points 1 to 2 in Figure 1). Since for a perfect gas the relation

(22) $$dS=C_{V}\frac{dT}{T}+R\frac{dV}{V}\;$$

can be applied, we observe that the constancy in entropy means that

(23) $$\frac{dT}{T}=-\frac{R}{c_{v}}\frac{dV}{V}$$

or by considering the value of c_v_ for the pure mono-atomic gas

(24) $$\frac{dT}{T}=-\frac{2}{3}\frac{dV}{V}$$

and by integration we obtain the simple relationship

(25) $$ln\frac{T_{1}}{T_{2}}=-\frac{2}{3}ln\frac{V_{1}^{\prime }}{V_{2}^{\prime }}=\frac{2}{3}ln\frac{V_{2}^{\prime }}{V_{1}^{\prime }}⟶\frac{T_{1}}{T_{2}}=e^{\frac{2}{3}}\;\frac{V_{2}^{\prime }}{V_{1}^{\prime }}\;$$

In other terms, during the adiabatic expansion, the increase in entropy due to the increase in volume of the piston expansion is compensated by an equivalent decrease in internal energy due to a decrease in temperature.

In summary, we have shown that the entropy conservation principle characteristic of the Carnot cycle can be interpreted in terms of a simple relationship between the volume of the gas and the temperature: during the two isothermal phases, the ratio between the involved final and initial volumes must be maintained; during the two adiabatic phases, the ratio between the involved initial and final temperatures leads to a direct relationship between the final and initial volumes.

Before moving on to the Carnot cycle treated according to information theory, we point out that positive and negative entropy contributions can also occur in out-of-equilibrium situations, as has been explicitly pointed out in the first part of this contribution [1] where the isothermal phase of the cycle is modified by “injections” of information coming from out-of-equilibrium situations. In [1] the use of the so-called “Jarzynski equality” [11] is cited in this regard, but positive and negative entropy contributions also occur in correspondence with fluctuations that occur in microscopic systems, as demonstrated by Y. Hua and Z-Y. Guo in [12] in the case of a thermoelectric cycle. In general, the *“entropy production fluctuation theorem”* can be kept in mind whenever one is dealing with microscopic systems that are kept far from equilibrium. In its most general formulation (see, for example, G.E. Crooks [13]), this theorem states that, for systems driven out of equilibrium by some time-dependent work process, $e^{\tau \sigma }=P\left(+\sigma \right)/P\left(-\sigma \right)\;$where $P\left(+\sigma \right)$ and $P\left(-\sigma \right)$ refer respectively to the positive and negative entropy production rates at time τ.

## 3. The Information Theory Carnot Cycle in the Light of the “Parametric Schrodinger Equations”

We have shown in the previous section how the systematic application of the two “Schrodinger equations” to the Carnot cycle allows the exploration of new aspects of the cycle itself. On the other hand, we have remarked in the Introduction that the Carnot cycle was the result of Carnot’s effort in understanding the steam machine, a complex thermodynamics system invented in order to transform heat into work. The possibility to apply the same “Schrodinger equations” in the context of information theory raises an obvious question: to whose “cycle” are they applied? Information theory was originated by Shannon in order to study a practical *communication system*, e.g., the telegraph and telephonic transmission. As shown in reference [2] Shannon’s first theorem (where he introduced the concept of capacity) was derived from Hartley’s studies on telegraph lines. Hence, we have to imagine a “communication cycle” where the information flux moves from a transmitter to a receiver and from the receiver to the transmitter under different impairment conditions. Shannon demonstrated that the impairments can be summarized as the unavoidable noise affecting the communication channel.

When Carnot modeled the cycle that bears his name, he never posed the “practical” problem of what the two heat reservoirs exactly were. In the same way we can imagine that, during communication, there are two “reservoirs” that act on the communication system whose variable, as we have already seen, is the “word length”. These reservoirs can “leave” information (e.g., noise enters into the channel) or can “lead” information, in any case perturbing the transmission.

**Figure 3:**
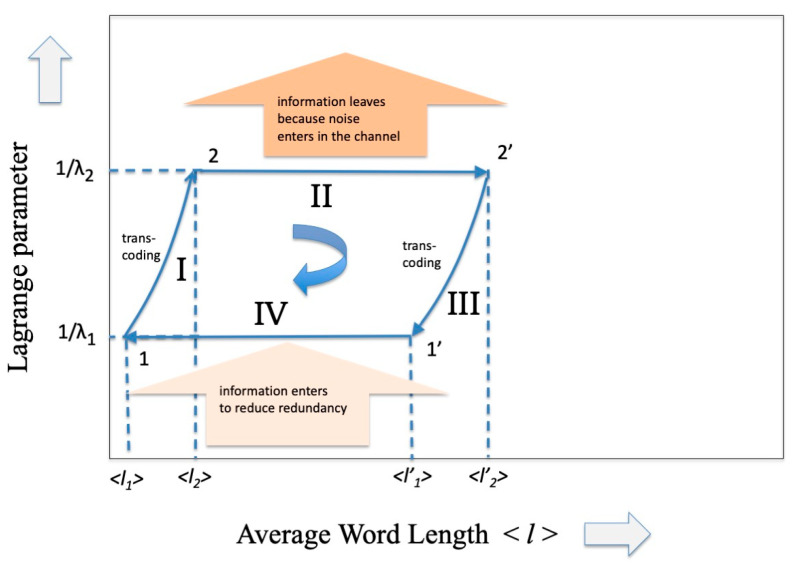
A representation of the Carnot Cycle in information theory that has as ordinate the inverse of the Lagrange parameter (related to the capacity of the communication channel) and as abscissa the average length of the codeword.

That is, we can imagine that, even in the scenario of information theory, there is a cycle as practical as that of the steam engine. The intent here is to communicate, that is, to “transport” information from a transmitter to a receiver, and vice versa, in the presence of a noise/information reservoir. The cycle closes by transporting the information in the opposite direction. Analogous to what happens in the cycle of the steam engine where two heat reservoirs at different temperatures are present, we could suppose that in this case the opposite transmission occurs at a different value of *channel capacity*, the parameter that the PSE sets equivalent to the inverse of the temperature. The cycle is illustrated in Figure 3 and will be analyzed below.

The cycle described above can be part of the category known in the literature as “information engines”. Among these “engines” one of the most interesting, in our opinion, is the one proposed on several occasions by T.S. Sundresh [14,15]. The author intends to *“explore information transactions between different entities comprising a system, as a unified way of describing the efficiency of integrated working on a system”* and examines this “engine” in *“analogy to the Carnot cycle”*. The author considers a very general system in which there are two “processes” and says: *“Two processes A and B are said to be integrated with each other when they can successfully cooperate with each other in doing a global task T”*, and he imagines that A and B process information and exchange information. The author then provides interesting examples of the global task T (of which we give only the title in the following for reasons of space): (a) two software modules working together in an integrated fashion to perform a computation; (b) organizational working in which information is transacted between various individuals or teams to work together on a given task; (c) scientific research environments; (d) human–machine interfaces; (e) computer-aided design. Then, introducing the methods of information theory and thermodynamics, the author draws a graph similar to that of Figure 1 with different meaning in the vertical coordinate.

We can therefore imagine that the cycle that will be described below occurs between two entities (in general, *“processes”* in the sense used by T.S. Sundresh) that behave both as receiver and transmitter, based solely on the need to complete the “task”.

Before proceeding, let us introduce some clarification in the PSEs that make them easier to manipulate. In information theory, the Boltzmann constant k equals 1 and a logarithm base 2 is adopted in order to measure the information in bits. Moreover, as recalled in the Introduction, the variable $\langle f\rangle$ as the expectation value of the codeword length $\langle l\rangle$ considered. Hence, (1) and (2) become

(26) $$\langle l\rangle =-\frac{d}{d\lambda }\log Z\left(\lambda \right)$$

(27) $$S=\log Z\left(\lambda \right)+\lambda \langle l\rangle$$

According to Jaynes [3], “the notion of word is meaningful only if there exists some rule by which a sequence of letters can be uniquely deciphered into words. If no such this rule exists then effectively each letter is a word”. Applied to codewords, the above sentence means that, when we consider all the codewords of the same length $\langle l\rangle =\log A$, the partition function becomes

(28) $$Z=\sum_{i=1}^{A}2^{-\lambda {\langle l\rangle }_{i}}=\sum_{i=1}^{A}2^{-\lambda lgA}=A2^{-\lambda }$$

Shannon’s first theorem defines “capacity” C as the eigenvalue of the above equation in the variable λ at the limit Z⟶1. Hence, for logZ=logA−λ⟶0, the capacity of the channel becomes

(29) $$\lambda =logA=C\;[\frac{bit}{symbol}]\;$$

In this way, a meaning is given to the Lagrange multiplier that becomes equal to the capacity of the channel (as mentioned by Jaynes: *”a given vocabulary may be regarded as defining a channel”*).

Having said this, let us start by asking ourselves: What happens when I inject noise (e.g., extract information) into the communication channel existing between the transmitter and the receiver? We know that in this case the error at the receiver inevitably increases because the receiver misinterprets the transmitter’s codewords. In order to restore correct transmission, it is necessary to use an appropriate coding technique (and Shannon’s theorems ensures that this is always possible) which increases the reliability of transmission (or, in information theory terms, reduces *equivocation*) at the cost of increasing *redundancy*.

In general, the *efficiency* of the code is defined as [16]

(30) $$\eta =\frac{H}{\langle l\rangle }$$

where *H* is the entropy of the source of the unit of symbols. The redundancy [17] is

(31) $$redundancy=1-\eta =\frac{\langle l\rangle -H}{\langle l\rangle }$$

and it is therefore observed how an increase in redundancy leads to an inevitable increase in the variable $\langle l\rangle$. A first example of redundancy comes from the coding technique itself. In fact, if we want to distinguish codewords, it is necessary to introduce some symbol between one word and another (for example, a space) and this causes redundancy. Jaynes [3] evaluates the expression of *H* in the case of a unique decodable code as

(32) $$H=log\langle l\rangle +\frac{\langle l\rangle -1}{\langle l\rangle }log\left(\frac{A-1}{\langle l\rangle -1}\right)=log\langle l\rangle +\frac{\langle l\rangle -1}{\langle l\rangle }log\left(A-1\right)-\frac{\langle l\rangle -1}{\langle l\rangle }log\left(\langle l\rangle -1\right)\;$$

That for large $\langle l\rangle$ becomes

(33) $$H\approx log\langle l\rangle +log\left(A-1\right)-log\left(\langle l\rangle \right)=log\left(A-1\right)\;$$

with efficiency

(34) $$\eta_{UD}=\frac{log\left(A-1\right)}{\langle l\rangle }$$

which is certainly lower than the case where there was no space character inserted.

Before proceeding, let us try to clarify the consequences of a change of the variable $\langle l\rangle$ (which is the parameter equivalent to the internal energy in the thermodynamics treatment). Since it is

(35) $$\langle l\rangle =\sum_{i}p_{i}l_{i}=\frac{1}{Z}\sum_{i}e^{-i\lambda l_{i}}l_{i}\;$$

we obtain

(36) $$d\langle l\rangle =\sum_{i}l_{i}{dp}_{i}+\sum_{i}p_{i}{dl}_{i}$$

where the first sum after the equals sign is in the equivalent position of “*change of heat, dQ*” which in our case can be a variation of the character probability and therefore we will call it *dP* and the second term in the equivalent position of “*negative change of work, −dW*” which in our case can be a variation of the codeword length and therefore we will call it *dK*, thus

(37) $$d\langle l\rangle =dP+dK$$

and, paraphrasing Tribus [17], we can say that “A change of the type *dK*, causes no change uncertainty S, since $p_{i}$ are unchanged. A change of the type *dP*, is associated with a change in S”.

Moreover, the distribution of $p_{i}l_{i}$ will represent the equivalent of the single-mode Planck’s law with a representation labeled with capacity instead of temperature.

When a communication system is subjected to noise, there is a direct impact on the mutual information defined as

(38) $$I\left(A;B\right)=H\left(A\right)-H\left(\frac{A}{B}\right)$$

where *A* and *B* are respectively the transmitter and receiver alphabet and the entropy $H\left(A/B\right)$ represents the *equivocation*

(39) $$H\left(A/B\right)=\sum_{B}p\left(a,b\right)log\frac{1}{p\left(a/b\right)}\;$$

where the probability $p\left(a/b\right)$ is the probability to detect b when a is sent and $p\left(a,b\right)$ is the joint probability. According to Abramson [16], *“the mutual information is equal to the average number of bit necessary to specify an input symbol before receiving and output symbol less the average number of bit necessary to specify and input symbol after receiving and output symbol”* and if the channel is not noiseless, “*the equivocation will not, in general, be zero, and each output symbol will contain only H(A) − H(A/B) bit of information*”. In order to restore the missing bits we therefore need codes that allow us to *correct errors* due to noise that occurs in the channel and in general these codes increase the redundancy of the communication and therefore increase the entropy.

For example, one of the first codes to be proposed [16] consists in repeating the message several times. However, repeating the message n times means considering the nth extension of the source, which reduces the transmitted information rate by n times. To avoid this, it can be shown that it is sufficient to use only *M* characters of the *n*th extension in order to keep the information rate *R*, defined as [16,18]

(40) $$R=\frac{logM}{n}$$

high enough. Together with Abramson [17] we can state that, since the nth extension of a source with r input symbols has a total of $r^{n}$ input symbols, if we use only *M* of these symbols, we may decrease the probability of error: *“The trick is to decrease the probability of error without requiring M to be so small that the message rate logM/n becomes too small. Shannon second theorem tell us that the probability of error can be made arbitrarily small as long as M is less than* $2^{nC}$ *and for this M the message rate becomes equal to the channel capacity* C=logM/n*”*.

So many codes have been invented that use the most varied mathematical algebraic properties and allow us to approach the limit of transmission capacity by paying a relatively modest price in terms of redundancy. A fairly recent review (2007) of these codes is reported in the fundamental paper by D.J. Costello and G.D. Forney [19] and more specifically for optical communications in a work by E. Agrell et al. [20]. In general, it is difficult to estimate the increase in redundancy in the case of error-correcting codes, given the very large variety of these. An average measure of this can be obtained by estimating the size of the overhead that must be inserted to reach the Shannon limit. For example, in 100 Gb/s coherent optical communication systems the overhead (or equivalently the $\langle l\rangle )\;$required to restore error-free transmission can be of the order of 20%. What can certainly be stated is that the introduction of these error-correcting codes perturbs the *dP* term in the expression (37) and therefore increases the entropy (27) as well as $\langle l\rangle$ (see Figure 3 from points 2 to 2′, where the input of noise is represented as “leaving the information”).

In summary, in phase II in the presence of noise (or “leaving of information”), the communication system presents an increase in entropy necessary to send the message from the transmitter to the receiver with the minimum of misunderstanding or with the maximum mutual information. In general, it is difficult to evaluate the increase in entropy and $\langle l\rangle \;$because it depends on the specific code used to reduce the error in the system.

What happens for the adiabatic phase of the cycle, i.e., phase III? In a thermodynamic framework we know that in this phase the temperature of the system *decreases,* entropy must be *conserved*, and heat exchange must be *zero*. As already underlined in the Conclusions of part I of this contribution, the interpretation of the “adiabatic” part for a communication system is quite complex. In fact, while in the isothermal cycle there is a reasonable parallel between the increase in entropy in the thermodynamic system and the parallel increase in entropy in the communication system, for the adiabatic part this similarity ceases. The decrease in temperature of the thermodynamic cycle translates into an equivalent increase in the Lagrange parameter which, according to the interpretation given in Equation (29), means relating to a communication system with greater capacity. Since we have assumed up to now that a transmission takes place in the presence of noise based on an alphabet A, we must now assume that at the receiver there is some device that takes care of translating the code from *A* to a richer alphabet *A′* while maintaining the same entropy value. In the cycle of Figure 1 we would have thus arrived at point 1′. To continue the cycle and to be able to “close” it, it is necessary at this point that the receiver transforms itself into a transmitter and transmits the message back to the previous transmitter (that now becomes a receiver) through a channel that, differently from phase II, *reduces* the entropy through an appropriate injection of information. Having arrived at point 1 it is easy to hypothesize at this point that there is another transcoder that brings the description of the message back to the original alphabet A, maintaining the same entropy (phase I), and so closing the cycle.

Let us follow the above hypothesis. From the analytical point of view, the change of the alphabet dimension leads to a change of the extreme of the partition function Z and of all the derived quantities. Hence, if we call this new alphabet A’, the entropy conservation means

(41) $$\log Z_{2}^{\prime }\left(A\right)+\lambda_{2}\langle l_{2}^{\prime }\rangle =\log Z_{1}^{\prime }\left(A\prime \right)+\lambda_{1}\langle l_{1}^{\prime }\rangle$$

where $\langle l_{2}^{\prime }\rangle$ is the average codeword length after the transmission and $\langle l_{1}^{\prime }\rangle$ is the average code length after the transcoding. If we make the reasonable working assumption that the error correction code used has restored almost all of the channel capacity and that transcoding occurs at full channel capacity, we can assume that both $Z_{2}^{\prime }$ and $Z_{1}^{\prime }$ in (41) are at the limit of 1 so that (41) becomes

(42) $$logA\langle l_{2}^{\prime }\rangle =logA\prime \langle l_{1}^{\prime }\rangle$$

thus

(43) $$\langle l_{1}^{\prime }\rangle =\frac{logA}{logA\prime }\langle l_{2}^{\prime }\rangle \;$$

As we expected, the new codewords will have a shorter average length because they refer to a larger alphabet. The effect of phase II on the codeword length is shown in Figure 3 (from point 2′ to 1′).

We are now at point 1′ of the cycle and in order to further reduce the average length $\langle l_{1}^{\prime }\rangle$ it is necessary to inject information in the system. As reported in [1] this can be performed by means of any “decision process” (see Ortega and Brown [21]) or more generally by means of any process that makes a selection, such as the selection processes that occur during the development of living beings (see, for example, B-O Kuppers [22]). Without repeating here what has already been demonstrated using the Kullback–Leibler distance in ref. [1] we may assume that $\langle l_{1}^{\prime }\rangle$ becomes $\langle l_{1}\rangle$ and, from point 1 with a further reverse transcoding, starting point 2 is reached and the loop is closed (see Figure 3). It is easy to observe analogies between Figure 2 and Figure 3: they show the same trend as regards the evolution of the “constant Lagrange parameter” phase and a similar trend (although symmetric) as regards the adiabatic zone. These analogies derive from the choice of representations in the two variables volume V and average codeword length $\langle l\rangle$, variables that come naturally when considering the practical realizations of the SPEs.

In summary, we have shown that, by using the variable codeword length $\langle l\rangle$, it is possible to trace a Carnot cycle even in information theory where the two isothermal phases are represented by a leaving and ingress of information while the two adiabatic phases are represented by transcoding operations. However, as already mentioned, the “volume” variable of the thermodynamic cycle and the “average codeword length” variable of the cycle in information theory emerge naturally when using PSEs to represent a classical Carnot cycle (where classical means the original one applied to heat engines) and an information theoretic cycle.

## 4. Conclusions

As we have seen in the previous two sections, PSEs are a powerful tool for analyzing the Carnot cycle and allow us to observe details that would not otherwise be visible. Although there is no direct parallel between the Carnot cycle in thermodynamics and information theory, there are some interesting analogies. Both frameworks can be represented with a diagram with coordinates of entropy/inverse of Lagrange parameter. From this diagram one can derive for the thermodynamic cycle the temperature/volume diagram and for the information theory cycle the capacity/average codeword length diagram.

In the thermodynamics framework, the area circumscribed by the cycle represents the work produced. It is in fact

(44) $$dW=dQ_{2}-dQ_{1}=T_{2}dS-T_{1}dS=\left(T_{2}-T_{1}\right)dS.$$

As far as the information theory framework is concerned, the same quantity is

(45) $$\left(\frac{1}{\lambda_{2}}-\frac{1}{\lambda_{1}}\right)dS.\;$$

that at the limit Z⟶1 yields

(46) $$\left(\frac{1}{logA}-\frac{1}{logA\prime }\right)dS$$

Since *A′* is a larger alphabet than *A* this means that a negative entropy is produced, thus information is gained by the Carnot information cycle. In other words, the transcoding operations makes the information injected in the presence of a larger alphabet heavier with respect to the redundancy accumulated in the presence of the smaller alphabet. Obviously, the cycle can also be built with the opposite direction, so producing noise.

Figure 3 and the previous observations constitute the answer to the question we started from: when the Carnot cycle is developed in the framework of information theory (a framework very far from the classical mechanical–thermal one) the conservation of entropy, that characterizes it, translates into a possible management of information that can be lost or gained. The analysis of this result and its consequences on complex systems that use information deserves further investigation.

## Data Availability

Data is contained within the article.

## Abbreviations

The following symbols are used in this manuscript:

A, B	transmitter and receiver alphabet
a, b	alphabet characters
A, A’	dimension of the considered ensemble/alphabet
C	channel capacity
$c_{v}$	specific heat at constant volume
$\epsilon_{i}$	energy of the gas molecules
η	code efficiency
$\eta_{UD}$	unique decodable efficiency
F	Helmholtz potential or free energy
$\langle f\rangle$	expectation value of $x_{i}$
H	entropy of the source
$\langle l\rangle$	average codeword length
λ	Lagrange multiplier
k	Boltzmann constant
M	character ensemble
P	gas pressure
$p_{i}$	probability of finding the system in the group index “i”
dQ	change of heat
S	entropy
T	Kelvin temperature
U	internal energy
V	gas volume
dW	change of work
Z	partition function
$x_{i}$	quality of the distinguishable group
